# Choline dihydrogen phosphate

**DOI:** 10.1107/S1600536809007259

**Published:** 2009-03-06

**Authors:** Kyoko Fujita, Douglas R MacFarlane, Keiichi Noguchi, Hiroyuki Ohno

**Affiliations:** a Department of Biotechnology, Tokyo University of Agriculture & Technology, 2-24-16 Naka-cho, Koganei, Tokyo 184-8588, Japan; b School of Chemistry, Monash University, Wellington Road, Clayton, VIC 3800, Australia; c Instrumentaion Analysis Center, Tokyo University of Agriculture & Technology, 2-24-16 Naka-cho, Koganei, Tokyo 184-8588, Japan

## Abstract

In the cystal structure of the title compound, (2-hy­droxy­ethyl)trimethylammonium dihydrogen phosphate, C_5_H_14_NO^+^·H_2_PO_4_^−^, two anions create dimeric structures *via* two O—H⋯O hydrogen bonds. The hydrogen-bonded dimers are connected by another O—H⋯O hydrogen bond with the hydroxyl groups of the cations, constructing a columner structure along the *a* axis. A number of C—H⋯O interactions are also present.

## Related literature

For background to ionic liquids, see: Byrne *et al.* (2007 ▶); Fujita *et al.* (2005 ▶); Ohno (2005 ▶); van Rantwijk *et al.* (2003 ▶); Seddon (1997 ▶); Wasserscheid & Welton (2002 ▶); Welton (1999 ▶); Zhao *et al.* (2008 ▶).
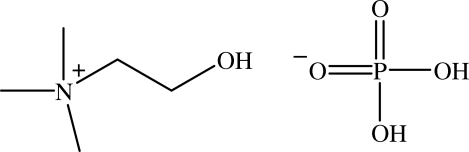


## Experimental

### 

#### Crystal data

C_5_H_14_NO^+^·H_2_PO_4_^−^
*M* *_r_* = 201.16
Triclinic, 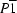

*a* = 6.9232 (3) Å
*b* = 8.2807 (4) Å
*c* = 9.2333 (3) Å
α = 84.458 (3)°
β = 71.414 (3)°
γ = 70.758 (3)°
*V* = 473.68 (4) Å^3^
*Z* = 2
Cu *K*α radiation
μ = 2.55 mm^−1^
*T* = 193 K
0.60 × 0.10 × 0.02 mm

#### Data collection

Rigaku RAXIS-RAPID diffractometer
Absorption correction: numerical (*NUMABS*; Higashi, 1999 ▶) *T*_min_ = 0.429, *T*_max_ = 0.950
8717 measured reflections
1714 independent reflections
1344 reflections with *I* > 2σ(*I*)
*R*_int_ = 0.053

#### Refinement

*R*[*F* ^2^ > 2σ(*F* ^2^)] = 0.042
*wR*(*F* ^2^) = 0.121
*S* = 1.12
1714 reflections
124 parameters
H atoms treated by a mixture of independent and constrained refinement
Δρ_max_ = 0.21 e Å^−3^
Δρ_min_ = −0.38 e Å^−3^

### 

Data collection: *PROCESS-AUTO* (Rigaku, 1998 ▶); cell refinement: *PROCESS-AUTO*; data reduction: *CrystalStructure* (Rigaku/MSC, 2004 ▶); program(s) used to solve structure: *SIR2004* (Burla *et al.*, 2005 ▶); program(s) used to refine structure: *SHELXL97* (Sheldrick, 2008 ▶); molecular graphics: *ORTEPIII* (Burnett & Johnson (1996 ▶); software used to prepare material for publication: *SHELXL97*.

## Supplementary Material

Crystal structure: contains datablocks global, I. DOI: 10.1107/S1600536809007259/at2730sup1.cif

Structure factors: contains datablocks I. DOI: 10.1107/S1600536809007259/at2730Isup2.hkl

Additional supplementary materials:  crystallographic information; 3D view; checkCIF report

## Figures and Tables

**Table 1:** Hydrogen-bond geometry (Å, °)

*D*—H⋯*A*	*D*—H	H⋯*A*	*D*⋯*A*	*D*—H⋯*A*
O3—H3*O*⋯O5^i^	0.80 (4)	1.79 (4)	2.586 (3)	178 (3)
O4—H4*O*⋯O2^i^	0.93 (4)	1.60 (4)	2.526 (2)	173 (3)
O5—H5*O*⋯O1^ii^	0.93 (4)	1.63 (4)	2.556 (3)	176 (4)
C3—H3*B*⋯O1	0.98	2.48	3.439 (3)	166
C4—H4*B*⋯O3^iii^	0.98	2.54	3.504 (3)	170
C4—H4*C*⋯O1^iv^	0.98	2.49	3.457 (3)	168
C5—H5*A*⋯O3^iv^	0.98	2.46	3.430 (3)	172
C5—H5*B*⋯O1^iii^	0.98	2.42	3.382 (3)	169
C5—H5*C*⋯O2	0.98	2.60	3.549 (3)	164

Symmetry codes: (i) 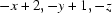; (ii) 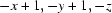; (iii) 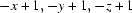; (iv) 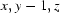.

**Fig. 1.**
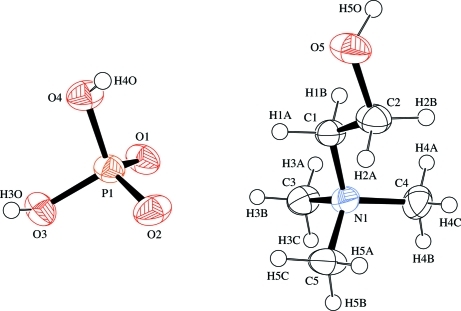
Displacement ellipsoid plot and atomic numbering scheme of (I). Ellipsoids are drawn at the 50% probability level and H atoms are shown as small spheres of arbitary radii.

**Fig. 2.**
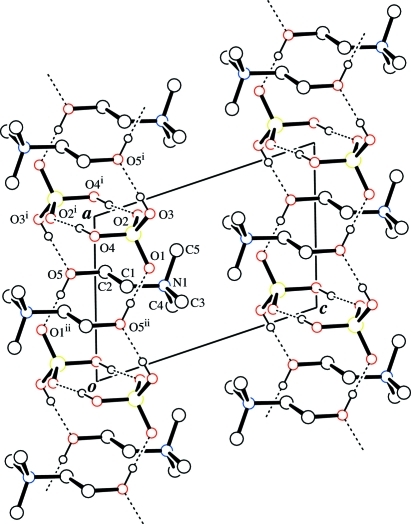
The molecular packing of (I) viewed along b axis. Dashed lines indicate intermolecular O—H···O hydrogen bonds. For clarity, only H atoms involved in O—H···O hydrogen bonding have been included. [Symmetry codes: (i) -x + 2, -y + 1, -z; (ii) -x + 1, -y + 1, -z.]

## supplementary crystallographic information

### Comment

Some ionic liquids (ILs) possess negligible vapor pressure as well as fascinating features such as high thermal, chemical and electrochemical stability. ILs have gained increasing attention as green, multi-use reaction media as well as solvents for a electrochemistry and chemistry (Welton, 1999; Seddon, 1997; Wasserscheid & Welton, 2002). ILs are also currently being investigated for a variety of bio-applications including media for biocatalytic reactions (van Rantwijk *et al.*, 2003; Zhao *et al.*, 2008), biosensors (Ohno, 2005) and protein stabilization (Fujita *et al.*, 2005; Byrne *et al.*, 2007). We have been studying hydrated IL as solvents for proteins. We have already reported that some proteins are soluble, stable, and remain active in some hydrated ILs. For example, the title compounds, acts as an excellent preserver of proteins such as cytochrome *c*.

The title compound (I) consists of cations and anions. The molecular structures of (I) are shown in Fig. 1. Two hydrogen bonds of O4—H···O2 connect anions and construct dimer along the *b* axis (Fig. 2). The dimers are connected with each other by the two hydrogen bonds of O5—H···O1 and O3—H···O5, through the hydroxyl group (Table 1). These hydrogen bonds create a columnar structure of anions and cations along the *a* axis. The columnar structures interact with each other by C—H···O hydrogen bond and van der Waals forces (Table 1).

### Experimental

Choline bromide solution was treated on an ion exchange resin (Amberlite IRN77), then mixed with phosphoric acid solution. The solvent evaporated and the product was dried *in vacuo*. White powder was dissolved in methanol, then reprecipited by dropping in acetone. This reprecipitation was repeated four times. Final purification was achieved by drowning-out crystallization from methanol solution. Aceton was used as antisolvent. This drowning-out crystallization was repeated twice at room temperature for X-ray measurements. The compound was identified using ^1^H NMR, DSC and Electrospray mass spectrometry.

### Refinement

The H atoms of the OH groups were found in difference maps and refined freely. The other C-bound H atoms were subsequently refined as riding atoms, with C—H = 0.98 and 0.99Å and *U*_iso_(H) = 1.2 or 1.5*U*_eq_(C).

### Crystal data

**Table:**

C_5_H_14_NO^+^·H_2_PO_4_^−^	*Z* = 2
*M**_r_* = 201.16	*F*(000) = 216
Triclinic, *P*1	*D*_x_ = 1.410 Mg m^−^^3^
Hall symbol: -P 1	Melting point: 392 K
*a* = 6.9232 (3) Å	Cu *K*α radiation, λ = 1.54187 Å
*b* = 8.2807 (4) Å	Cell parameters from 6930 reflections
*c* = 9.2333 (3) Å	θ = 5.1–68.3°
α = 84.458 (3)°	µ = 2.55 mm^−^^1^
β = 71.414 (3)°	*T* = 193 K
γ = 70.758 (3)°	Platelet, colourless
*V* = 473.68 (4) Å^3^	0.60 × 0.10 × 0.02 mm

### Data collection

**Table:**

Rigaku RAXIS-RAPID diffractometer	1714 independent reflections
Radiation source: rotating anode	1344 reflections with *I* > 2σ(*I*)
graphite	*R*_int_ = 0.053
Detector resolution: 10.00 pixels mm^-1^	θ_max_ = 68.3°, θ_min_ = 5.1°
ω scans	*h* = −8→8
Absorption correction: numerical (*NUMABS*; Higashi, 1999)	*k* = −9→9
*T*_min_ = 0.429, *T*_max_ = 0.950	*l* = −11→11
8717 measured reflections	

### Refinement

**Table:**

Refinement on *F*^2^	Primary atom site location: structure-invariant direct methods
Least-squares matrix: full	Secondary atom site location: difference Fourier map
*R*[*F*^2^ > 2σ(*F*^2^)] = 0.042	Hydrogen site location: difference Fourier map
*wR*(*F*^2^) = 0.121	H atoms treated by a mixture of independent and constrained refinement
*S* = 1.12	*w* = 1/[σ^2^(*F*_o_^2^) + (0.0626*P*)^2^ + 0.050*P*] where *P* = (*F*_o_^2^ + 2*F*_c_^2^)/3
1714 reflections	(Δ/σ)_max_ < 0.001
124 parameters	Δρ_max_ = 0.21 e Å^−^^3^
0 restraints	Δρ_min_ = −0.38 e Å^−^^3^

### Special details

**Table:**

Geometry. All e.s.d.'s (except the e.s.d. in the dihedral angle between two l.s. planes) are estimated using the full covariance matrix. The cell e.s.d.'s are taken into account individually in the estimation of e.s.d.'s in distances, angles and torsion angles; correlations between e.s.d.'s in cell parameters are only used when they are defined by crystal symmetry. An approximate (isotropic) treatment of cell e.s.d.'s is used for estimating e.s.d.'s involving l.s. planes.
Refinement. Refinement of *F*^2^ against ALL reflections. The weighted *R*-factor *wR* and goodness of fit *S* are based on *F*^2^, conventional *R*-factors *R* are based on *F*, with *F* set to zero for negative *F*^2^. The threshold expression of *F*^2^ > σ(*F*^2^) is used only for calculating *R*-factors(gt) *etc*. and is not relevant to the choice of reflections for refinement. *R*-factors based on *F*^2^ are statistically about twice as large as those based on *F*, and *R*- factors based on ALL data will be even larger.

### Fractional atomic coordinates and isotropic or equivalent isotropic displacement parameters (Å^2^)

**Table:**

	*x*	*y*	*z*	*U*_iso_*/*U*_eq_	
P1	0.81645 (9)	0.68990 (7)	0.17447 (6)	0.0335 (2)	
O1	0.5777 (2)	0.7534 (2)	0.24514 (18)	0.0430 (5)	
O2	0.9220 (3)	0.50241 (19)	0.19693 (17)	0.0439 (5)	
O3	0.9039 (3)	0.8007 (2)	0.25187 (19)	0.0390 (4)	
O4	0.8798 (3)	0.7343 (2)	0.00080 (18)	0.0406 (4)	
O5	0.7017 (3)	0.2172 (2)	−0.10979 (19)	0.0452 (5)	
N1	0.4405 (3)	0.2917 (2)	0.3125 (2)	0.0340 (5)	
C1	0.5122 (4)	0.3227 (3)	0.1424 (2)	0.0342 (5)	
H1A	0.5978	0.4020	0.1227	0.041*	
H1B	0.3832	0.3801	0.1096	0.041*	
C2	0.6444 (4)	0.1635 (3)	0.0453 (2)	0.0391 (6)	
H2A	0.7750	0.1035	0.0755	0.047*	
H2B	0.5596	0.0844	0.0584	0.047*	
C3	0.3093 (4)	0.4616 (3)	0.3894 (3)	0.0417 (6)	
H3A	0.1821	0.5104	0.3547	0.050*	
H3B	0.3958	0.5391	0.3633	0.050*	
H3C	0.2640	0.4464	0.5003	0.050*	
C4	0.3029 (4)	0.1774 (3)	0.3487 (3)	0.0458 (7)	
H4A	0.1836	0.2257	0.3057	0.055*	
H4B	0.2458	0.1681	0.4597	0.055*	
H4C	0.3893	0.0636	0.3042	0.055*	
C5	0.6299 (4)	0.2169 (3)	0.3699 (3)	0.0435 (6)	
H5A	0.7117	0.1024	0.3255	0.052*	
H5B	0.5805	0.2089	0.4815	0.052*	
H5C	0.7219	0.2902	0.3403	0.052*	
H3O	1.025 (5)	0.793 (4)	0.209 (3)	0.058 (9)*	
H4O	0.959 (6)	0.643 (5)	−0.067 (4)	0.096 (12)*	
H5O	0.597 (5)	0.232 (4)	−0.158 (4)	0.090 (12)*	

### Atomic displacement parameters (Å^2^)

**Table:**

	*U*^11^	*U*^22^	*U*^33^	*U*^12^	*U*^13^	*U*^23^
P1	0.0331 (4)	0.0388 (4)	0.0280 (4)	−0.0104 (3)	−0.0088 (3)	−0.0016 (2)
O1	0.0311 (9)	0.0607 (12)	0.0372 (9)	−0.0139 (8)	−0.0102 (7)	−0.0032 (8)
O2	0.0622 (11)	0.0359 (10)	0.0292 (9)	−0.0108 (8)	−0.0130 (8)	0.0003 (7)
O3	0.0323 (9)	0.0490 (10)	0.0363 (9)	−0.0148 (8)	−0.0066 (7)	−0.0093 (7)
O4	0.0474 (10)	0.0392 (10)	0.0298 (9)	−0.0082 (8)	−0.0099 (7)	−0.0009 (7)
O5	0.0372 (9)	0.0728 (13)	0.0290 (9)	−0.0221 (9)	−0.0102 (7)	0.0022 (8)
N1	0.0408 (11)	0.0326 (10)	0.0299 (10)	−0.0123 (8)	−0.0125 (8)	0.0028 (8)
C1	0.0372 (12)	0.0396 (13)	0.0284 (12)	−0.0143 (10)	−0.0124 (10)	0.0042 (9)
C2	0.0421 (13)	0.0476 (14)	0.0282 (12)	−0.0154 (11)	−0.0103 (10)	0.0001 (10)
C3	0.0476 (14)	0.0366 (13)	0.0340 (12)	−0.0063 (11)	−0.0097 (11)	−0.0028 (10)
C4	0.0574 (16)	0.0469 (15)	0.0356 (13)	−0.0284 (13)	−0.0064 (11)	0.0038 (11)
C5	0.0506 (15)	0.0435 (14)	0.0352 (13)	−0.0045 (12)	−0.0223 (11)	0.0006 (11)

### Geometric parameters (Å, °)

**Table:**

P1—O1	1.4969 (16)	C1—H1A	0.9900
P1—O2	1.5080 (16)	C1—H1B	0.9900
P1—O4	1.5629 (16)	C2—H2A	0.9900
P1—O3	1.5771 (17)	C2—H2B	0.9900
O3—H3O	0.79 (3)	C3—H3A	0.9800
O4—H4O	0.93 (4)	C3—H3B	0.9800
O5—C2	1.427 (3)	C3—H3C	0.9800
O5—H5O	0.93 (4)	C4—H4A	0.9800
N1—C5	1.493 (3)	C4—H4B	0.9800
N1—C4	1.499 (3)	C4—H4C	0.9800
N1—C3	1.499 (3)	C5—H5A	0.9800
N1—C1	1.513 (3)	C5—H5B	0.9800
C1—C2	1.513 (3)	C5—H5C	0.9800
			
O1—P1—O2	115.19 (10)	C1—C2—H2A	110.3
O1—P1—O4	110.63 (9)	O5—C2—H2B	110.3
O2—P1—O4	110.24 (9)	C1—C2—H2B	110.3
O1—P1—O3	104.81 (9)	H2A—C2—H2B	108.6
O2—P1—O3	109.78 (10)	N1—C3—H3A	109.5
O4—P1—O3	105.63 (10)	N1—C3—H3B	109.5
P1—O3—H3O	113 (2)	H3A—C3—H3B	109.5
P1—O4—H4O	117 (2)	N1—C3—H3C	109.5
C2—O5—H5O	114 (2)	H3A—C3—H3C	109.5
C5—N1—C4	110.68 (19)	H3B—C3—H3C	109.5
C5—N1—C3	108.80 (19)	N1—C4—H4A	109.5
C4—N1—C3	108.66 (19)	N1—C4—H4B	109.5
C5—N1—C1	110.65 (17)	H4A—C4—H4B	109.5
C4—N1—C1	110.51 (18)	N1—C4—H4C	109.5
C3—N1—C1	107.44 (16)	H4A—C4—H4C	109.5
N1—C1—C2	114.88 (18)	H4B—C4—H4C	109.5
N1—C1—H1A	108.5	N1—C5—H5A	109.5
C2—C1—H1A	108.5	N1—C5—H5B	109.5
N1—C1—H1B	108.5	H5A—C5—H5B	109.5
C2—C1—H1B	108.5	N1—C5—H5C	109.5
H1A—C1—H1B	107.5	H5A—C5—H5C	109.5
O5—C2—C1	107.09 (19)	H5B—C5—H5C	109.5
O5—C2—H2A	110.3		
			
C5—N1—C1—C2	62.5 (3)	C3—N1—C1—C2	−178.9 (2)
C4—N1—C1—C2	−60.5 (3)	N1—C1—C2—O5	−178.51 (17)

### Hydrogen-bond geometry (Å, °)

**Table:**

*D*—H···*A*	*D*—H	H···*A*	*D*···*A*	*D*—H···*A*
O3—H3O···O5^i^	0.80 (4)	1.79 (4)	2.586 (3)	178 (3)
O4—H4O···O2^i^	0.93 (4)	1.60 (4)	2.526 (2)	173 (3)
O5—H5O···O1^ii^	0.93 (4)	1.63 (4)	2.556 (3)	176 (4)
C3—H3B···O1	0.98	2.48	3.439 (3)	166
C4—H4B···O3^iii^	0.98	2.54	3.504 (3)	170
C4—H4C···O1^iv^	0.98	2.49	3.457 (3)	168
C5—H5A···O3^iv^	0.98	2.46	3.430 (3)	172
C5—H5B···O1^iii^	0.98	2.42	3.382 (3)	169
C5—H5C···O2	0.98	2.60	3.549 (3)	164

Symmetry codes: (i) −*x*+2, −*y*+1, −*z*; (ii) −*x*+1, −*y*+1, −*z*;  (iii) −*x*+1, −*y*+1, −*z*+1; (iv) *x*, *y*−1, *z*.
